# Elaborated pollen packaging and dispensing mechanism induced by petal architecture from a Papaveraceae species

**DOI:** 10.7717/peerj.7066

**Published:** 2019-06-11

**Authors:** Shanlin Yang, Guangming Chu, Xiang Shi, Shaoming Wang

**Affiliations:** 1 College of Agriculture, Shihezi University, Shihezi, China; 2 College of Life Sciences, Shihezi University, Shihezi, China

**Keywords:** Pollination process, Secondary pollen presentation, Middle lobe, *Hypecoum erectum* L., Elaborate petals, Packaging and dispensing mechanism

## Abstract

Secondary pollen presentation (SPP) is a reproductive strategy that enhances the efficiency of pollen transfer, which has been explored for more than 200 years, resulting in 10 identified types of SPP. The ephemeral plant *Hypecoum erectum* L. (Papaveraceae) has an elaborate petal structure. The middle lobe is a key functional organ in SPP. To explore the importance of the middle lobe structure, we measured the flowering process, the curling movement and growth of the middle lobe, pollination characteristics, pollination efficiency, and the mating system in *H. erectum* in the field. The yellow middle lobe structure had an important role in attracting pollinators. The middle lobes on the inner petals function as a redundant cucullate structure and wrapped about 84% of the total pollen grains as soon as the anthers dehisced. These then grew upward and gradually presented pollen to pollinators via the roll out of the middle lobes. One bee species, *Colletes vestitus* from Colletidae, was the only effective pollinator of *H. erectum*. The SPP mechanism increased the efficiency of pollen transfer by *C. vestitus*. The middle lobes, which wrapped pollen and grew upward, contacted the stigma and provided an advantage for self-pollination and outcrossing by growing upward higher than the corolla. *Hypecoum erectum* L. has a mixed mating system with selfing and outcrossing. Thus, the SPP mechanism plays a key role during the pollination process and is necessary for improving pollination efficiency and promoting reproductive success.

## Introduction

Most flowers are hermaphrodites, obtaining fitness by both dispersing pollen (male function) and receiving pollen (female function) (Fan, Kress & Li, 2015). For some animal-pollinated flowers, pollen reception can be sufficient with only a few visits by pollinators, however, the reproductive success by pollen dispersal usually continues to increase with numerous visits by pollinators (Bell, 1985). Accordingly, there are stronger selective pressures for the male function than the female function in the flower’s characteristics. In order to improve the male function, the pollen presentation mechanisms which can promote pollen transfer will be favored (Harder & Wilson, 1994; Wilson et al., 1994). Plants use dispersing mechanisms or pollen packaging mechanisms in order to disperse pollen to pollinators (Lloyd & Yates, 1982). Packaging mechanisms allow the total pollen production of a plant to be distributed into separate parts in order to continually become available to pollinators. Plants can control the presentation speed and presentation time of pollen with the packaging mechanism (Lu & Tan, 2007). Dispensing mechanisms limit the available pollen that can be removed by a pollinator from the package in a single visit (Lloyd & Yates, 1982). The effective packaging and/or dispensing strategies could allow all of the pollinators visiting a flower to take part in pollen dispersal, while ensuring the removal of most of the pollen produced by the time flowering is complete (Harder & Thomson, 1989).

Petals are a characteristic feature of eudicots and the most noticeable organs of the flower. They have evolved into a great variety across diverse clades. Color, morphology, size, structure, and the function of petals reveal a broad diversity over evolutionary time and the elaborate petals mainly present as tubular, bilabial, cup-shaped, flat, marginal lobes, and ventral lobes of various shapes (Irish, 1998; Endress & Matthews, 2006; Zhao, 2016; Thien et al., 2009). A variety of lobation patterns can play a crucial part in strengthening the floral structure (Schönenberger, 1999), attracting pollinators (Thien & Marcks, 1972), guiding pollinators to the nectar (Rogers, 2004), promoting self-pollination (Liu et al., 2008), and secondary pollen presentations (SPPs) (Yeo, 1993). The middle lobes, as part of the lobation pattern, can be adapted to the lateral lobes and become important petal structures (Moody & Hufford, 2000). The middle lobe in *Chenorchis* has a plane structure across which pollinators can move and which is also an important structure for self-pollination in some plants (Liu et al., 2008). Endress & Matthews (2006) analyzed 58 species of plants with special petal characteristics in 30 families and found that the lobation patterns in 11 families were closely related to the evolution of floral characteristics. However, there is limited research on the evolution of lobation patterns, especially the middle lobes (Riviere, Clayson & Dockstader, 2013).

Secondary pollen presentation (SPP) is the phenomenon where pollen is presented to pollinators on floral structures (other than the anthers) or via particular mechanisms of expulsion where pollen contacts other floral structures (Yeo, 1993). Secondary pollen presentation was first described by Sprengel (1793) in *Campanula*. However, for a long time afterwards, this phenomenon has been neglected in the study of floral characteristics. International studies of pollen presentation schedules in the evolution of angiosperms have mainly focused on the past 30 years. Concurrently, some researchers have explored the evolution of floral characteristics from the perspective of the pollinator’s adaptations, so that the pollen presentation theory can be formed and continuously improved (Wang & Tan, 2011). The presentation of pollen on a surface other than the primary pollen presentation organ (anthers) may protect pollen from desiccation or exploitation, aid in cross-pollination or self-pollination, and increase the efficiency of pollen delivery (Yeo, 1993). Attracted pollinators and the enhanced male function of the flowers of SPP plants is obvious via the extension of the male phase by the protection, controlled release, and clear placement and receipt of the pollen (Howell, Slater & Knox, 1993). This phenomenon has only been recorded in 25 families, including five monocotyledonous and 20 dicotyledonous families (Fan, Kress & Li, 2015). There are many interesting SPP mechanisms not yet discovered in nature.

As one of the main plant families with elaborate petal structures, the Papaveraceae have been studied since the 19th century. Elaborate floral characteristics mainly occur in some genera of *Dicentra*, *Corydalis*, and *Hypecoum*. *Hypecoum* species, however, have received very little research attention. The SPP function in *Hypecoum*, which is enhanced by the middle lobe structure, was first described and illustrated by Hildebrand in 1869. Hildebrand (1869), however, rarely observed pollinators visiting flowers and did not record stigma receptivity. Dahl (1989) found that median anthers in a single flower in *Hypecoum* are vascularized by two bundles and the transversal anthers by one. Although the above scholars have made many groundbreaking discoveries on the *Hypecoum*, many details related to its pollination characteristics and breeding system have not been discussed.

*Hypecoum erectum* L. is a typical ephemeral plant and is widely distributed throughout the Gurbantunggut Desert in Xinjiang, China. The *H. erectum* flower is bisymmetric and characterized by the elaborate shape of the two inner petals. The inner petals are deeply trilobate with yellow middle lobes. The aim of this study was to research the floral characteristics and explore how the middle lobes improve male fitness of *H. erectum*. We proposed the following hypotheses: *Hypecoum erectum* L. may have a SPP mechanism because of the relative position of the middle lobes and stamens, and the process of gradual pollen presentation in the middle lobes plays an important role in increased pollination efficiency because the length of the pollen presentation time and visitation frequencies will be increased. In light of the above hypotheses, we investigated the floral characteristics and pollination adaptability of *H. erectum*. The study of such elaborate petal structures in *H. erectum* will be helpful in understanding the adaptations of SPP and the role of reproductive success in floral evolution.

## Materials and Methods

### Study site and species

All experiments were carried out in 2017 at the Cainan oilfield (45°02′22″ N, 88°23′26″ E; 728 m a.s.l; 100 m × 100 m), which is on the southeastern edge of the Gurbantunggut Desert in the Xinjiang Uyghur Autonomous Region, China. Periodically, steady snow in this region creates an opportunity for species to germinate. In 2017, there was steady snow cover for 100–160 days, which was 20–30 cm deep in the winter. According to the data from the nearest weather station, the Fukang National Field Scientific Observation and Research Station for Desert Ecosystems, the mean annual temperature was 8.4 °C, and the mean temperatures of the coldest and the hottest months were −15.7 °C in January and 26.1 °C in July, respectively. The mean annual precipitation was 220 mm, but the mean annual evaporation was 10 times the precipitation amount (Zhang et al., 2006). The snow in winter and the rainwater in spring provided favorable conditions for ephemeral plants to grow (Wang et al., 2004).

**Figure 1:**
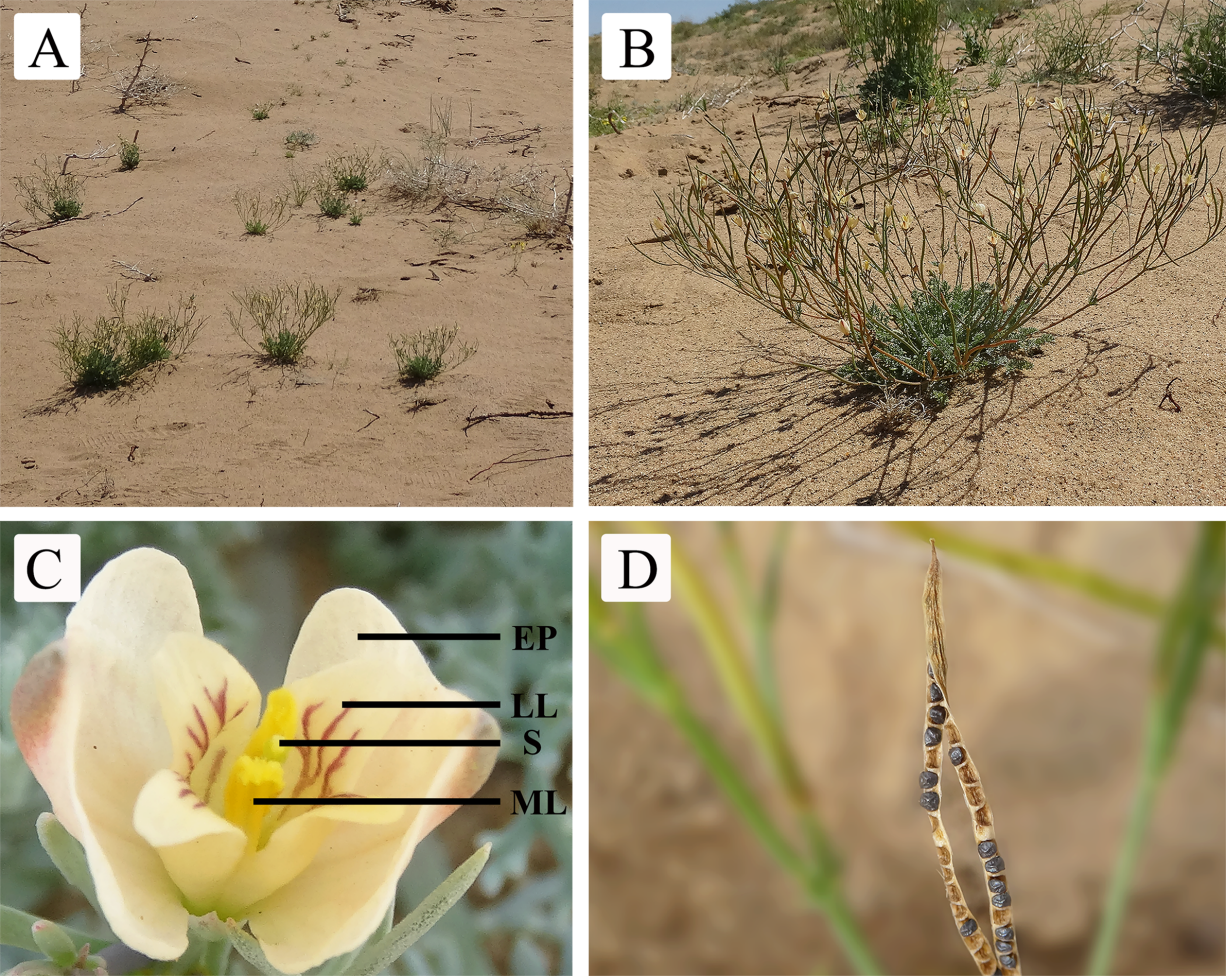
Population, individual, single flower, and morphological characteristics of *H. erectum*. (A) Population. (B) Individual plant. (C) Flower characteristic. EP, external petal; LL, lateral lobe; S, stigma; ML, middle lobe. (D) Capsule with seeds showing longitudinal dehiscence.

*Hypecoum erectum* L. is an ephemeral plant in the desert. From October to November, the seeds in the seed bank start to germinate and grow and form the autumn-germinated seedlings. These seedlings spend the winter under the snow and continue to complete their life cycle into the spring of the second year. March and April typically correspond with the period for spring-germinated seedlings. The autumn-germinated plants and spring-germinated plants complete their life cycle in June. *Hypecoum erectum* L. has a patchy distribution (Fig. 1A). Individual plants are 13.54 ± 1.24 cm tall (Fig. 1B). Inflorescences are dichasial cymes (Fig. 1B). Petals are white and yellow, external petals are obovate, and the inner petals consist of lateral lobes and middle lobes; the lateral lobes have red or purple veining and the middle lobes are yellow (Fig. 1C). Each flower has one pistil and the androecium consists of four stamens. The flower of *H. erectum* is dichogamy and protogyny; Anthers are yellow. The fruits of *H. erectum* are capsules with linear and with a longitudinal dehiscence (Fig. 1D). The data in this paper were collected from April to June, 2017.

### Floral syndrome

#### Observation of flowering dynamics

About 30 plants were randomly selected from a larger population for flowing dynamic observation. The number of flowers per dichasial cymes was statistically calculated to be 90 inflorescences. There were 270 flower buds of an average size that were randomly selected in order to record the opening time of the flowers, the opening direction of the flowers, floral longevity, and the time of anther dehiscence. Data were recorded daily on 90 flowers and with replications every 3 days. Ten plants were selected from the 30 plants to record the relative height between anthers, stigma, and middle lobes among the four flowering phases. Four flowers were separately selected with the four flowering phases from one plant.

#### Observation of the curling movement and growth of the middle lobe

To explore how pollen was wrapped and dispersed by the middle lobes, the curling movement and growth of the middle lobes was investigated. In the morning, there were 20 flower buds of an average size from 10 individuals that were randomly selected (two flower buds per individual) and divided into two groups, with 3 days replications. Each group contained 10 flower buds and was exposed to one of two conditions: (1) natural conditions; or (2) bagged and screened from pollinators. We recorded the curling movement speed and the movement and growth processes of the middle lobes. Weather conditions were recorded in order to detect if there were differences in the curling movement and growth of the middle lobes.

### Pollination characteristics

#### Strategy of pollen allocation

To examine the strategy of pollen allocation, 10 flower buds of the same average size from 10 plants were bagged with paper bags to screen pollinators. The next day, after the middle lobes grew upward and were equal in length to the stigma, each 10 middle lobes, anthers, corollas, and stigmas were removed from the flowers and fixed in glass vials for pollen counts. Another 10 flower buds with the same average size from 10 plants were collected and taken back to the laboratory. Before anther dehiscence, 40 anthers were cut off and loaded into glass vials for pollen counts. The pollen size was photographed and measured under a scanning electron microscope (JSM-6490LV; JEOL, Akishima, Japan).

All the anthers were immersed in one ml of 10 % HCl solution to dissolve the anther walls and dilute with distilled water to 10 mL. A micropipette was used to transfer five μL of the solution to microscope slides. The numbers of pollen in each sample were counted under an optical microscope (Olympus BH-2, Olympus, Tokyo, Japan). The process was repeated 10 times.

#### Attraction of floral characteristics to pollinators

To investigate the importance of elaborate floral characteristics on attracting pollinators, 120 single flowers in the peak flowering phase were divided into 12 groups, each containing 10 flowers, for four different pollination treatments, with three replicates: (1) control; (2) without lateral lobes; (3) without middle lobes; and (4) without stamens. The pollinator visitation rate was observed and recorded on sunny days continuously during the peak pollinator visitation period from 12 pm to 2 pm for 3 days in the peak flowering phase.

#### Observation of the pollination process

Three *H. erectum* plants were observed on three sunny days in May (May 19–21). Pollinators that made effective contact with the anthers and/or stigma, foraging behaviors, foraging time, retention time, and sequentially visited flower numbers were continuously observed between 8 am and 9 pm each day from a distance of one m. To analyze the optimum temperature and wind speed for pollination, both were recorded at 1-h intervals using an anemograph (AZ-8901, Taiwan, China). The floral display size was noted at the end of each interval. Hourly visitation frequency was calculated by dividing the number of pollinator visits per plant per hour by the number of floral displays per hour.

All visiting pollinators were collected and put into glass bottles containing diethyl ether. Pollen on the pollinators’ bodies was washed onto slides using 70% ethanol and examined under an optical microscope at 100× magnification. Pollinators were considered effective if (i) they carried *H. erectum* pollen on their body and (ii) they contacted either the anthers or the stigma during the visit. After observation, specimens were sent for identification to the Institute of Zoology, Chinese Academy of Sciences.

#### Statistics of pollen removal

According to the Harder (1990) method, the pollen removal was tested by deducting the pollen remaining for a visited flower from the total pollen production by an unvisited flower or anther on the same plant. To explore the pollination function of the middle lobes, pollen removal of two experimental manipulations in two foraging behaviors were estimated by the amount of pollen taken away after a single visit of a pollinator to a single flower. We chose 20 plants with the same basic crown and bearing not fewer than two inflorescent branches as experimental plants for manipulation experiments. There were 60 flower buds with the same average size selected from the 20 plants (three flower buds per plant), and the number of pollen grains in each flower bud was counted and analyzed for the differences in the number of the pollen grains in flower buds among the 20 plants. Then another 40 flower buds with a similar size from the same 20 plants were marked and bagged (two flower buds per plant), and divided in two groups: (i) 30 flower buds from 15 plants were the control. (ii) 10 flower buds from five plants were cut to remove the two middle lobes. The next day, the bag was removed from each newly opened flower for a single visit. Upon one visitation, the foraging behavior was recorded, we chose 10 flowers visited by foraging behavior I, 10 flowers visited by foraging behavior II, and 10 flowers without middle lobes. All the floral structures were carefully removed and fixed in ethanol separately to count the number of pollen grains. We also analyzed the differences in the number of the remained pollen grains in each group of five plants after a single visit by a pollinator.

### Observation of the mating system

To determine the mating system, 360 flower buds from more than 90 plants were randomly selected. Four flower buds were selected from each plant and then one of four pollination treatments were carried out. Each treatment contained 30 flower buds and with three replications. These treatments were: (1) natural pollination; (2) retention of four stamens and bagging; (3) emasculation, outcrossing, and bagging; (4) supplemental outcrossing. Flowers were hand pollinated with the pollen from other plants at least 100 m away. All emasculation treatments were carried out before anthers dehiscence. Pollinators were excluded using paper bags. Fruits were collected 30 days after manipulation, fruit set and seed set were calculated. Seeds from supplemental outcrossing, selfing, and outcrossing were placed in 90-mm-diameter Petri dishes with two layers of filter paper and with eight mL of distilled water to moisten the filter paper. Four replications of 40 seeds of each Petri dishes were used per treatment. All the Petri dishes were placed in an incubator in 24 h darkness for 30 days at 25/10 °C (optimum conditions). The germination of seeds was not counted until the end of the incubation.

### Data analysis

In this study, all statistical analyses were performed using SPSS 17.0 software. Figures were prepared using Origin8.5 software. Data were represented as means ± standard errors. The differences among the four groups (control, no stamens, no middle lobes, and no lateral lobes) were examined using paired-sample *t*-tests. The differences among the two foraging behaviors in the number of pollen grains dropped in the corolla in a single visit was examined using a paired-sample *t*-test. One-way ANOVA was used to analyze the differences in the number of the pollen grains in 60 flower buds among the 20 plants, to analyze the differences in the number of the remaining pollen grains in each group after a single visit by a pollinator, and to compare the pollination treatments effects.

## Results

### Floral syndrome

#### Flowering dynamic observations

Peak flowering occurred in mid-May in 2017. Inflorescences had 6–14 flowers. Flowers persisted for 1–2 days, the opening time of (91.11 ± 0.64%) the flowers occurred mainly between the hours of 9.30 am to 10.30 am, while the others chose to open at other times of the day. The opening direction of the flowers was upward on sunny days, but those that opened on rainy days were directed downward. On rainy days, the duration of flowering was prolonged by 1–2 days compared to sunny days.

**Figure 2:**
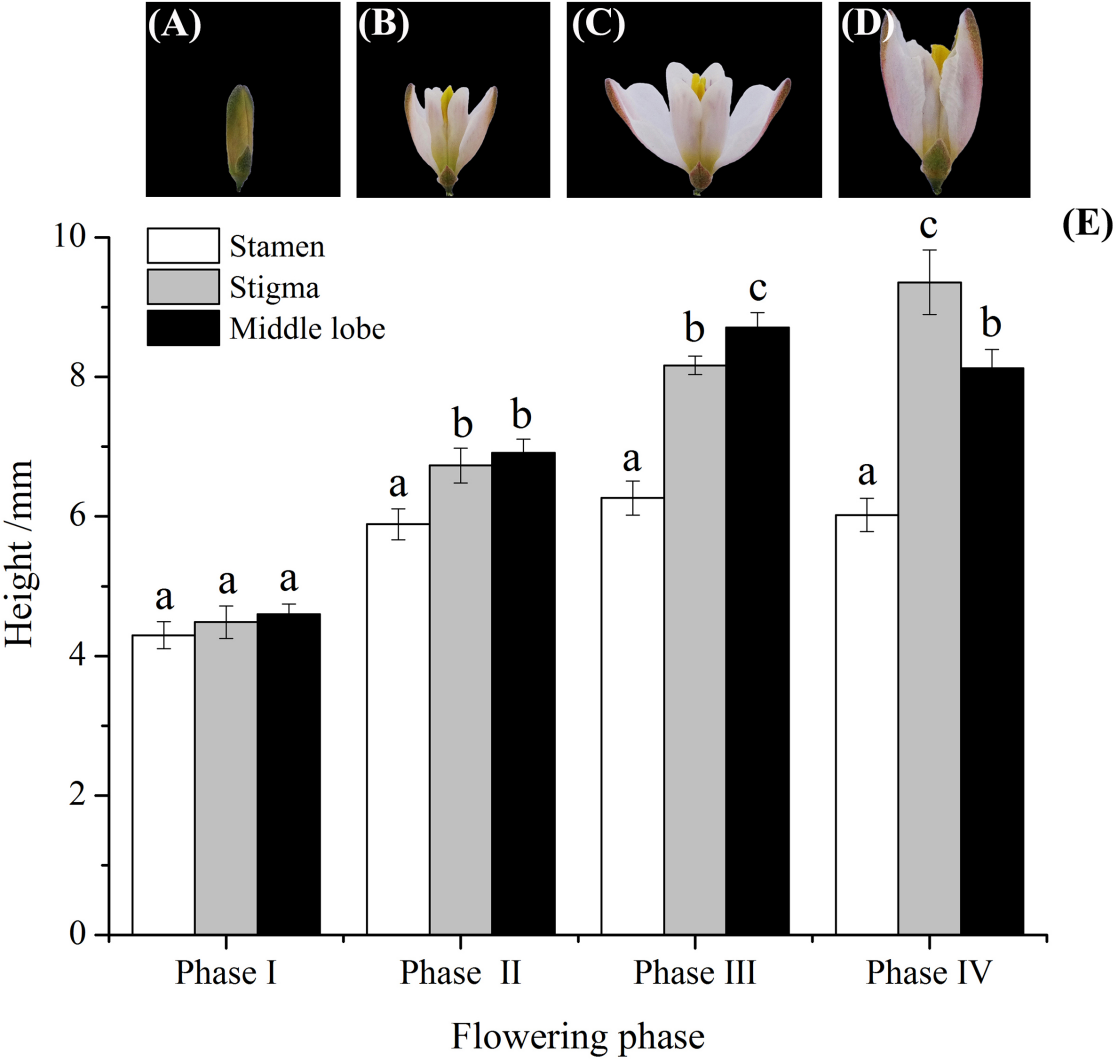
Flowers variation, and relative height among stamens, stigma, and middle lobes of *H. erectum*’s flower. (A) Anther had not dehisced; (B) anther had dehisced; (C) middle lobes started to curl movement; (D) middle lobe completed the growth and curl movement; (E) relative height among stamen, stigma, and middle lobe. Different lowercase letters indicate significant differences among the same phase at *p* < 0.05 levels.

The flowering span was divided into four phases according to whether or not anthers had dehisced and on the curling movement and growth of middle lobes, the flowering span was divided into four phases (Figs. 2A–2D). Anthers dehisced around noon after the flowers opened. Then, middle lobes wrapped pollen from one and two half anthers, folded up, and grew upward until they were equal in length to the stigma, and could be used for self-pollination (Fig. 2E, phase II). Petals fully opened at phase III, and the middle lobes were significantly taller than the pistils and stamens (Fig. 2E, phase III).

#### Progress of SPP

**Figure 3:**
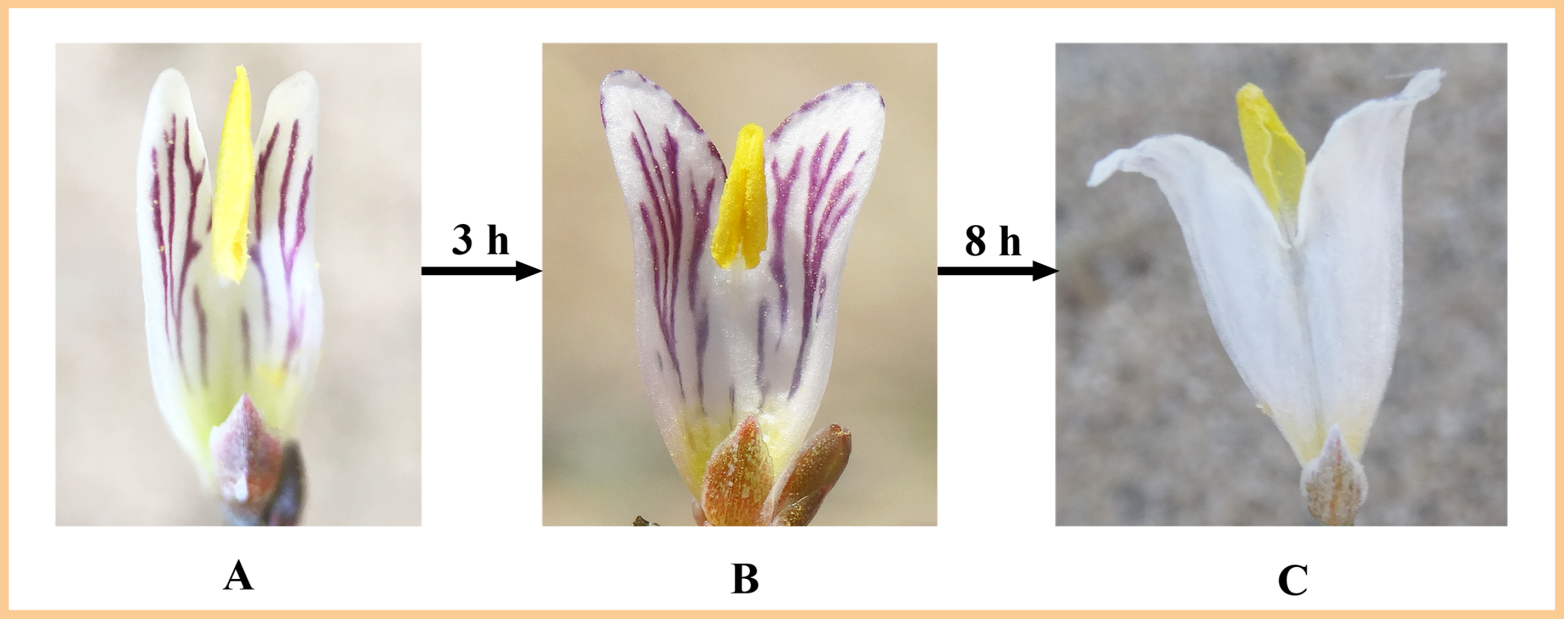
The process of secondary pollen presentation in *H. erectum*. (A) After anthers dehiscence, most of the pollen grains were wrapped in the pouched middle lobe. (B) Three hours after middle lobes wrapped pollen, the middle lobe started to curl outward from the edge to form a gap and release a part of the pollen. (C) Eight hours after flowers start curling outward, the middle lobe is deflexed backward and releases all the pollen (the picture shows the back of the inner petal).

On a sunny day, the anthers dehisced around noon after flowers opened. At the anther dehiscence, the two middle lobes on two inner petals were functioned as a SPP structure. The middle lobes wrapped pollen as soon as the anthers dehisced, obtained pollen, and grew upward a bit to contact the stigma, thus allowing selfing (Fig. 3A). The height of the middle lobes was similar to the stigma. Then the middle lobes continued to grow upward to conspicuous position to achieve outcrossing. About 3 h after the flowers opened, at about 12.30 pm to 1:20 pm, the middle lobes started to curl outward from the edge to form a gap and present pollen from the gap (Fig. 3B). The cucullate middle lobe structure was the key functional organ in SPP. Approximately 8 h after the flowers curled outward, at about 8.30 pm to 9.30 pm, the middle lobes were deflexed backwards until they became convex, and thus pollen was available to pollinators (Fig. 3C). The strategy of SPP in *H. erectum* is for a gradual pollen presentation. On a rainy day or with bagging to screen pollinators on a sunny day, the flowering span of single flowers was prolonged.

### Pollination characteristics

#### Strategy of pollen allocation

Under natural conditions, the total number of pollen grains in one flower was 74,160 ± 1,283. The pollen grains were about 19.312 ± 0.412 μm in diameter. After anthers dehiscence, the average number of pollen grains on the stigma, four anthers, and corolla were 2,880 ± 174, 6,480 ± 252, and 2,540 ± 181, respectively, while the number of pollen grains presented on the two middle lobes was 62,520 ± 1,544, i.e. about 84% of the total pollen grains.

#### Attraction of the floral characteristics to pollinators

**Figure 4:**
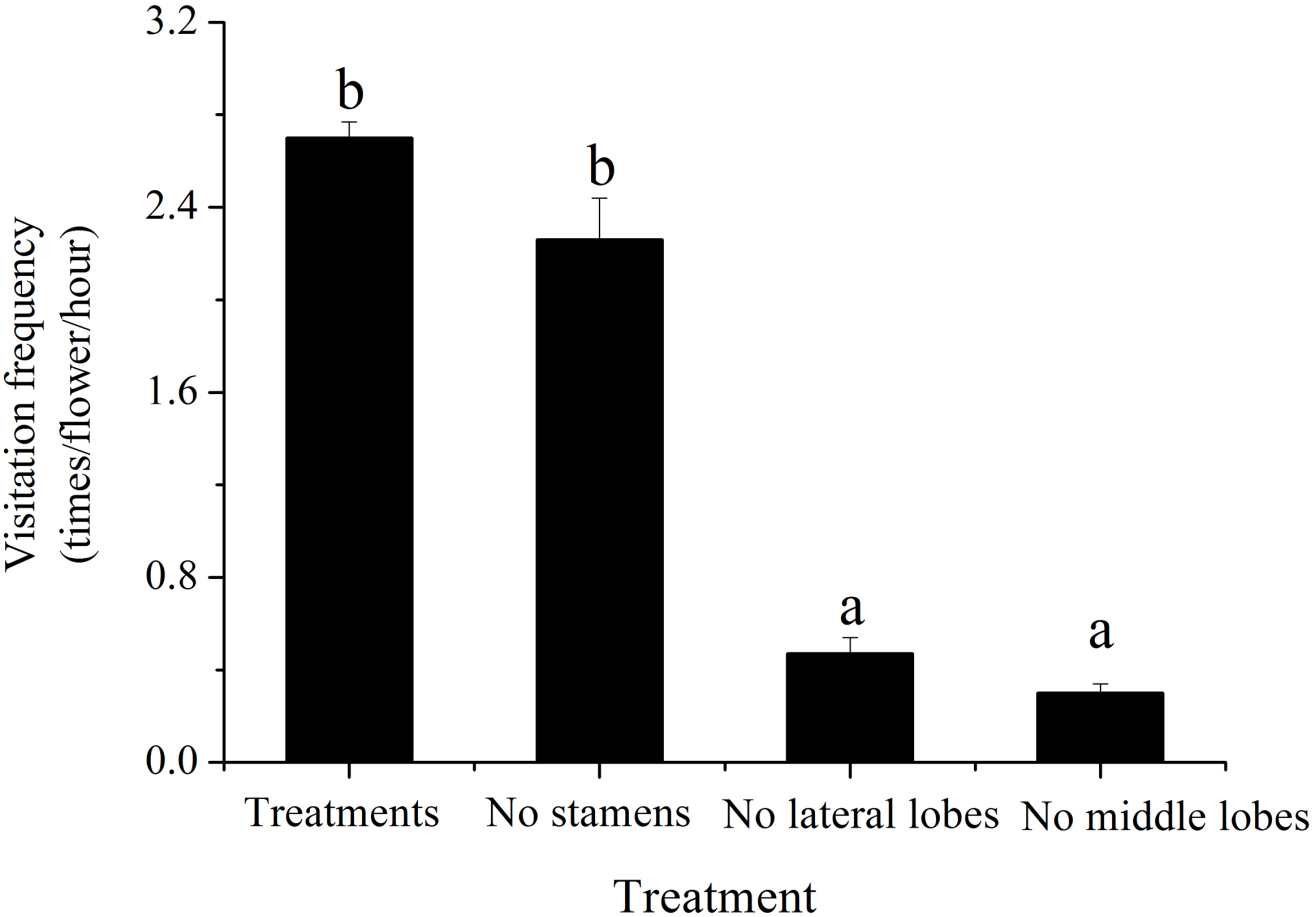
Effect of reduced flower attraction by removal of flower parts on pollinator visitation frequency of *H. erectum*. Different lowercase letters indicate significant differences among different treatments at *p* < 0.05 levels.

Compared to control treatments, the number of visiting pollinators was significantly lower when the middle lobes or lateral lobes were cut (*p*_*M*_ = 0.000; *p*_*L*_ = 0.000) (Fig. 4). Cutting out two middle lobes had a more significant effect on reducing the number of visiting pollinators (nearly one-ninth of the natural state). There was no significant difference in attracting pollinators by removing four stamens (*t* = 2.365, *p* = 0.142).

#### Pollinator observation

**Figure 5:**
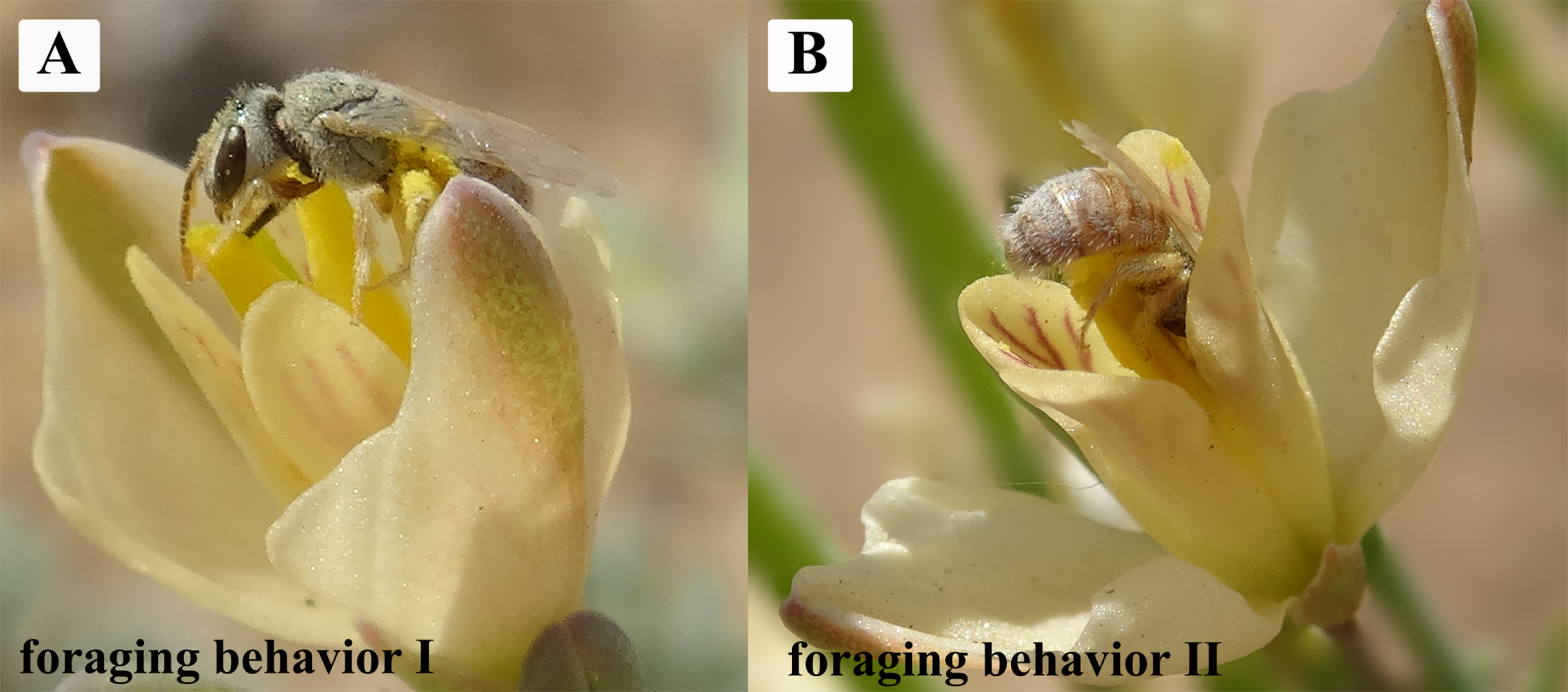
The principle and only effective pollinator of *H. erectum*: *Colletes vestitus* (Colletidae) showing the two reported forms of foraging behavior. (A) Foraging behavior I: pollinator collects the pollen from the closed middle lobes. (B) Foraging behavior II: pollinator sticking its body into the corolla to suck nectar when middle lobe deflexed.

**Figure 6:**
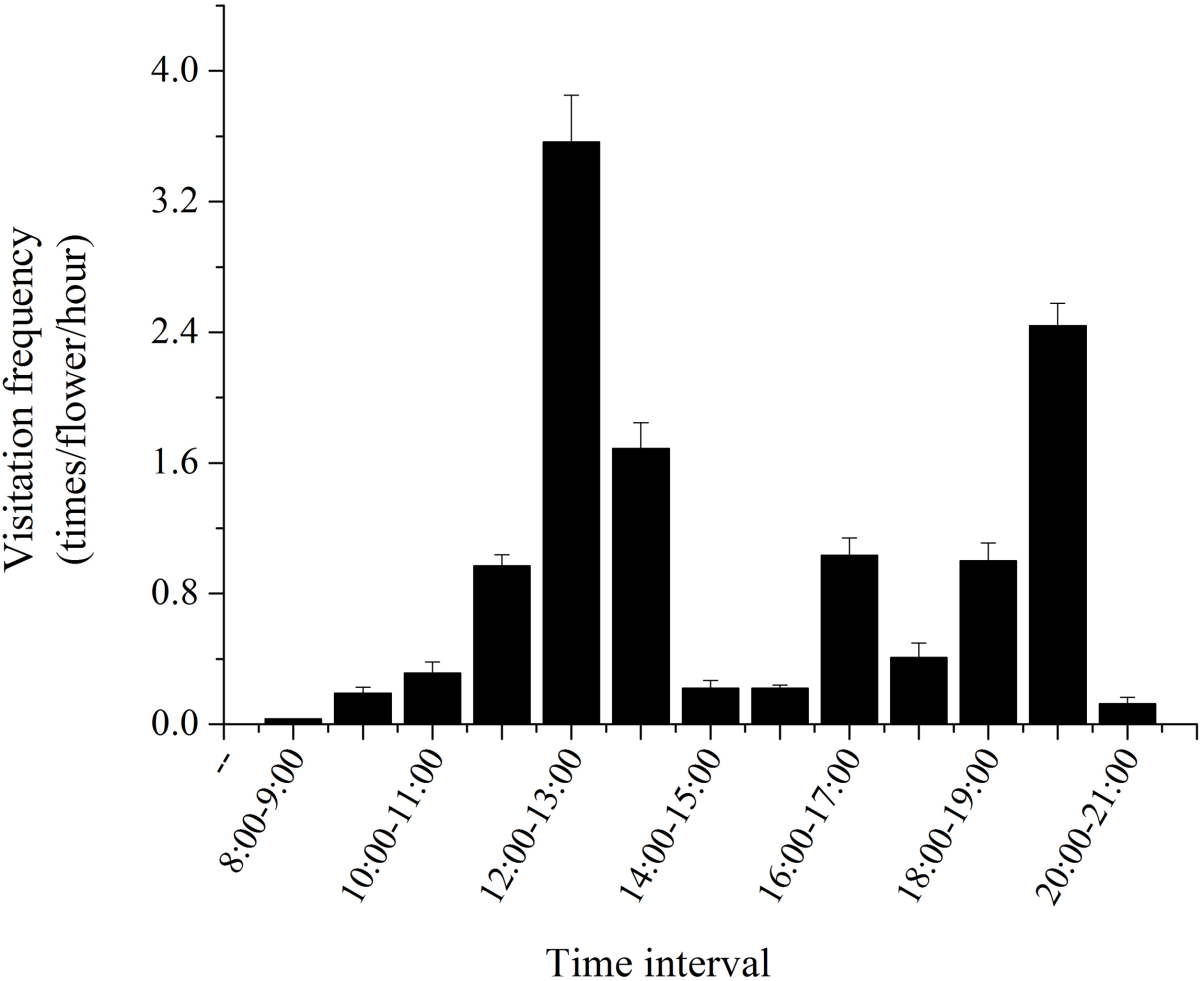
Daily dynamics of the visitation frequency of the only effective pollinators of *H. erectum*.

Our studies showed that *H. erectum* was pollinated exclusively by Colletidae bees. *Colletes vestitus* was the only effective pollinator in the natural population of *H. erectum* (Figs. 5A and 5B). The average visiting frequency of pollinators was 12.19 ± 1.34 visits/flower/day. Average residence time of pollinators on a flower was 22.35 ± 1.25 s. Pollinators sequentially visited three to four different flowers on the same plant in a single visit. Peak visitation occurred at 12 pm to 1 pm and 7 pm to 8 pm (Fig. 6). The temperature and wind speed had important effects on foraging behaviors. The optimum temperature for pollinators was approximately 16–32 °C. When the wind speed was higher than 3.5 m/s, there was no effective pollinator in any population. After landing on a flower, pollinators behaved differently when obtaining pollen. A total of 1,170 pollinator visits were recorded over 3 days of observation, with 351 visits categorized as foraging behavior I and 819 visits as foraging behavior II.

Foraging behavior I (where the edge of middle lobes was closed): *C. vestitus* landed on one of the two middle lobes, and “unzipped” it (i.e., from top to bottom) to open another middle lobe from the closed edge with its proboscis. Then, the bee continuously obtained pollen from the middle lobe using its forefoot when there was a gap on the middle lobe by unzipping many times and continually grooming pollen grains into their corbiculae (Fig. 5A). Pollinators used their head and/or thorax to touch the stigma.

Foraging behavior II (where the edge of middle lobes was opened and curled outward): *C. vestitus* stuck its head into the corolla and used its proboscis to take up nectar at the base of the ovary. At the same time, the head and thorax contacted the residual pollen on anthers (Fig. 5B). During the process of moving up and down, the bee’s abdomen and corbiculae contacted pollen that was presented on the curled outward middle lobes and the stigma.

#### Pollination efficiency and pollen removal

Our results showed that there was no significant difference in the number of pollen grains in 60 flower buds among the 20 plants (*F*_60 single flowers_ = 0.361, *p* = 0.990). There was no significant difference in the number of the remaining pollen grains in each group of five plants after a single visit by a pollinator (*F*_foraging behavior I_ = 2.041, *p* = 0.227; *F*_foraging behavior II_ = 0.328, *p* = 0.849; *F*_without middle lobes_ = 3.254, *p* = 0.114). When the middle lobes were retained, most pollen grains remained in the middle lobes, and less pollen was present on the corolla and anthers after either of a single visit by the two foraging behaviors; the pollen grains that remained in middle lobes could be dispersed to pollinators continuously by the gradual pollen presentation. The amount of pollen remaining in the corolla was significantly higher in flowers visited using foraging behavior I than in flowers collected using foraging behavior II (*t* = 10.590, *p* = 0.000) (Table 1). When the middle lobes were artificially cut, all the pollen grains that remained on the anthers were presented to pollinators simultaneously and visited by pollinators directly. More than 11.84% of the total pollen grains (8,780 ± 267) were dropped and groomed inside the corolla after the anther dehiscence and a single visit by pollinators and could not be removed by pollinators.

**Table 1:** The number of pollen grains removed from different flower organs by pollinators in different foraging behaviors.

Project	Middle lobes retained		Middle lobes removed
	After one visitation by foraging behavior I	After one visitation by foraging behavior II	After one visitation by pollinator
The number of pollen grains removed from anthers	\	800 ± 90	9,860 ± 586
The number of pollen grains removed from middle lobes	7,180 ± 301	5,300 ± 369	\
The number of pollen grains removed from stigma	700 ± 70	1,320 ± 78	\
The number of pollen grains removed from corolla	\	\	\
The number of pollen grains dropped in the corolla	2,320 ± 231	880 ± 131	5,680 ± 252
The number of pollen grains remained in middle lobes	55,340 ± 301	57,220 ± 369	\
The number of pollen grains remained in corolla	4,860 ± 198	3,420 ± 144	8,780 ± 267
The number of pollen grains remained in anthers	6,480 ± 252	5,680 ± 90	61,380 ± 586
The number of pollen grains remained in stigma	2,180 ± 70	1,560 ± 78	\

### Determined to mating system type

*Hypecoum erectum* L. exhibited a mixed mating system (Table 2). Fruit set established with no emasculation and bagging was 78.89 ± 0.011%, which showed that spontaneous selfing usually happened. Artificial outcrossing produced the highest seed set, which indicated that it preferred outcrossing. Aside from the seed set and germination rate from supplemental outcrossing, both were significantly higher than that from selfing (*t*_seed set_ = −14.966, *p* = 0.004; *t*_germination rate_ = 5.468, *p* = 0.012), which indicated that outcrossed pollens might be preferentially selected when self and outcross pollen grains are both found on the stigma. The results also revealed that inbreeding depression plays a role in the seed set and germination.

**Table 2:** Fruit set, seed set and germination rate (means ± SE) of *H. erectum* were compared between each pollination treatment.

Treatments	Natural pollination	No emasculation and bagging	Emasculation, outcrossing and bagging	Supplemental outcrossing
Fruit set (%)	81.11 ± 0.022^a^ (*N* = 90)	78.89 ± 0.011^a^ (*N* = 90)	80.00 ± 0.019^a^ (*N* = 90)	82.22 ± 0.029^a^ (*N* = 90)
Seed set (%)	80.62 ± 0.004^b^ (*N* = 90)	72.74 ± 0.015^a^ (*N* = 90)	87.58 ± 0.009^c^ (*N* = 90)	80.71 ± 0.01^b^ (*N* = 90)
Germination (%)	50.63 ± 3.44^b^ (*N* = 160)	32.50 ± 2.70^a^ (*N* = 160)	59.38 ± 4.13^b^ (*N* = 160)	53.75 ± 3.15^b^ (*N* = 160)

**Note:**

Different lowercase letter indicates significant differences among the different treantments at the same level at *p* < 0.05 levels.

## Discussion

Our study described a mechanism of SPP in a natural population of *H. erectum* in Papaveraceae. We found that about 84% of the total pollen grains were presented on the two middle lobes (the SPP organ) through the wrapping up of middle lobes after the anthers dehiscence. This mechanism has a selective advantage and about 16% of the remaining pollen grains were simultaneously presented on the anthers, stigma, and corolla. We found that the SPP mechanism of *H. erectum* promoted the efficiency of pollinators to remove pollen. As a gradual pollen presentation strategy, this SPP mechanism probably evolved under selection via the male function.

### The yellow middle lobe structure is an important petal structure for attracting pollinators

Animal-pollinated flowering plants often generate spectral signals to suit the color capabilities of the important pollinators or other potential pollinators in the environment (Shrestha et al., 2013). Many flowers have yellow floral guides and bright yellow spots on the petals to attract pollinators (Shangguan et al., 2008; Lunau et al., 2017). Our results showed that when cut off, the yellow middle lobes significantly reduced the frequency with which *C. vestitus* visited *H. erectum*, and caused many pollen grains to be groomed into the corolla, making them unusable. The attractive flowers are likely to be more successful at transferring pollen to other plants (Snow, 1982). The yellow middle lobe of *H. erectum* played an important role in effectively attracting pollinators, promoting pollen transport, and improving pollination efficiency.

### The middle lobe is a key organ in SPP

In angiosperms, plants potentially compete to transport donated pollen to receptive stigmas (Castellanos et al., 2006). The specific mechanisms for pollen presentation are geared towards the maximization of the number of effective pollinator visits to individual flowers (Weigend, Ackermann & Henning, 2010). Lebuhn & Holsinger (1998) concluded that, “A plant should allocate pollen such that all pollinators that visit remove pollen”. Between the *Penstemon* and *Keckiella* species, anthers vary in ways that can affect the pollen release, and the morphology of the dried anthers shows how they dispense pollen (Castellanos et al., 2006). However, the plant’s success as a father may depend on its temporal and spatial deployment of pollen (Lloyd & Yates, 1982) because some allocations of pollen among pollinators may reduce the uncertainty of successful pollen transport, thereby promoting pollen dispersal to the stigma. Flowers can package pollen in different floral structures and dispense pollen to pollinators in different spaces or time. The middle lobes of *H. erectum*, as the SPP organ, took up 84% of the total pollen grains from the anthers after the anthers dehiscence, then folded up, and dispersed pollen to pollinators in portions. In *H. erectum*, two floral characteristics were key for SPP function: (1) the relative position of the anthers to the middle lobe, and (2) the curling movement and growth of the middle lobe. The former enables pollen to be wrapped by middle lobes, while the latter promotes the presentation of more pollen by gradual pollen presentation. The middle lobe structure played a crucial role in the evolution of the SPP mechanism in *H. erectum*.

### The indispensable role of middle lobes in pollen export and pollination efficiency

*Hypecoum* species have unique floral traits, making them the ideal phylotype for studying pollination in flowering plants (Fedde, 1990). To determine how floral characteristics promote pollen distribution in a small range within a pollinator’s body and pollen distribution through various body sites of a pollinator, research should be focused on correlations between the pollination mechanism and the flowering pattern of plants (Huang et al., 2014). In *H. erectum*, after the anthers dehiscence, pollen was presented both on the anthers and on the middle lobes, enabling pollinators to remove pollen simultaneously from both middle lobes and anthers during a single visit. Secondary pollen presentation promoted pollen removal compared with the primary pollen presentation, where pollen is solely presented on the anthers. In addition, pollen is deposited on the abdomens of bees simultaneously, which can enhance the value of the pollinator (Lloyd, 1984). This arrangement allows *H. erectum* to effectively use the pollinator’s abdomen for pollination and further promotes pollen removal.

Most flowering plants depend on animals for pollination. Animal pollination likely results in diminishing returns through varied mechanisms, including pollinator grooming, a limited capacity to carry pollen, layering of pollen on the pollinator, and local mate competition (Harder & Wilson, 1994; Harder & Johnson, 2008). Owing to the activities of pollinators, the pollen loss during the process of pollen transfer is usually high and usually <1% of the pollen removed from anthers is able to reach conspecific stigmas (Harder & Wilson, 1994; Lu & Tan, 2007). In this case, limiting the pollen uptake by pollinators could help increase the fitness of the males. Therefore, many plants present their pollen in small doses rather than all at once to maximize the amount of pollen delivered to stigmas. In the Campanulaceae, all the anthers release pollen on the outside surface of the stigma, where the pollen presentation is controlled by the retractable hairs on the stigma. The progress of retraction occurs successively along the stigma and limits the pollen removal by a single visit of pollinators (Lloyd & Yates, 1982, Harder & Thomson, 1989; Thomson, Wilson & Valenzuela, 2010). In the present study, about 84% of the total pollen grains were wrapped in the middle lobes and gradually exposed to pollinators when the middle lobes were curled outside. This feature indicated that the pollen presentation strategy of *H. erectum* was gradual pollen presentation. Flowers with gradual pollen presentation can effectively limit the number of pollen grains taken away from a single flower by pollinators each time, prolonging pollen presentation time and enlarging the pollen presentation area. This strategy will attract more pollinators to join in the pollination process, increasing the chance of pollen contribution and improving male fitness (Lu & Tan, 2007). Therefore, the SPP mechanism of *H. erectum* reduced pollen loss and improved pollination efficiency, which can be adaptive.

The two foraging behaviors revealed differences in *H. erectum*. During a single visit, bees using foraging behavior I removed more pollen from the middle lobes than bees using foraging behavior II, but foraging behavior I led to more pollen being dropped into the corolla than foraging behavior II. Although foraging behavior I had higher waste, it was still a valuable pollinator behavior. Pollination is generally dependent on the pollinator effectiveness of each visit and the visitation rate per individual pollinator (Waser et al., 1996). In *H. erectum*, the visitation numbers of foraging behavior I accounted for 30% of the total visits. The results indicated that: (1) both foraging behaviors were able to increase the plant fitness after one visit, enabling pollen removal and seed production; and (2) foraging behavior I removed more pollen grains from the middle lobes than foraging behavior II in a single visit, but the pollen removal efficiencies were higher in foraging behavior II than in foraging behavior I. Therefore, the successful pollination of *H. erectum* is a result of an interplay between foraging behavior I and foraging behavior II.

## Conclusions

The SPP mechanism of *H. erectum* plays an important role in successful pollination. The middle lobe on the two inner petals is specialized as a cucullate structure. The middle lobe will wrap the anther as soon as the anther dehisces, obtaining pollen, and growing upward. The cucullate structure is the key functional organ in attracting pollinators and in SPP. The results showed that cutting the yellow middle lobe significantly reduced the visitation frequency and increased waste. Furthermore, the middle lobe plays a key role in attracting pollinators, removing pollen, and improving male fitness. These results are in agreement with the suggestion made by Howell, Slater & Knox (1993) that the SPP may lengthen the male phase and increase the efficiency of pollen transfer.

## Supplemental Information

10.7717/peerj.7066/supp-1 Supplemental Information 1 Population, individual, single flower, and morphological characteristics of *H. erectum*. (A) Population. (B) Individual plant. (C) Flower characteristic. EP, external petal; LL, lateral lobe; S, stigma; ML, middle lobe. (D) Capsule with seeds showing longitudinal dehiscence.

10.7717/peerj.7066/supp-2 Supplemental Information 2 Flowers variation, and relative height among stamens, stigma, and middle lobes of *H. erectum’s* flower. (Note: Different lowercase letters indicate significant differences among the same phase at *p* < 0.05 levels).

10.7717/peerj.7066/supp-3 Supplemental Information 3 The process of secondary pollen presentation in *H. erectum*. (A) After anthers dehiscence, most of the pollen grains were wrapped in the pouched middle lobe; (B) Three hours after middle lobes wrapped pollen, the middle lobe started to curl outward from the edge to form a gap and release a part of the pollen; (C) Eight hours after flowers start curling outward, the middle lobe is deflexed backward and releases all the pollen (the picture shows the back of the inner petal).

10.7717/peerj.7066/supp-4 Supplemental Information 4 Effect of reduced flower attraction by removal of flower parts on pollinator visitation frequency of *H. erectum*. (Note: Different lowercase letters indicate significant differences among different treatments at *p* < 0.05 levels).

10.7717/peerj.7066/supp-5 Supplemental Information 5 The principle and only effective pollinator of *H. erectum*: *Colletes vestitus* (Colletidae) showing the two reported forms of foraging behavior. (A) Foraging behavior I: pollinator collects the pollen from the closed middle lobes; (B) Foraging behavior II: pollinator sticking its body into the corolla to suck nectar when middle lobe deflexed.

10.7717/peerj.7066/supp-6 Supplemental Information 6 Flowering dynamic and the movement speed of the middle lobes including the number of the flowers per inflorescence, flower longevity, opening time of the flowers, and the movement time of the middle lobes.

10.7717/peerj.7066/supp-7 Supplemental Information 7 Daily dynamics of the visitation frequency of the only effective pollinators of *H. erectum*.

10.7717/peerj.7066/supp-8 Supplemental Information 8 The number of pollen grains removed from different flower organs by pollinators in different foraging behaviors.

10.7717/peerj.7066/supp-9 Supplemental Information 9 Fruit set, seed set, and germination rate (means ± SE) of *H. erectum* were compared between each pollination treatment.

## Additional Information and Declarations

### Data Availability

The following information was supplied regarding data availability: The raw measurements are available in the Supplemental Files.
