# Supplementary figures and images for: Elaborated pollen packaging and dispensing mechanism induced by petal architecture from a Papaveraceae species
Source: PeerJ. 2019 Jun 11;7:e7066. doi: 10.7717/peerj.7066 (PMC6568250; doi:10.7717/peerj.7066)

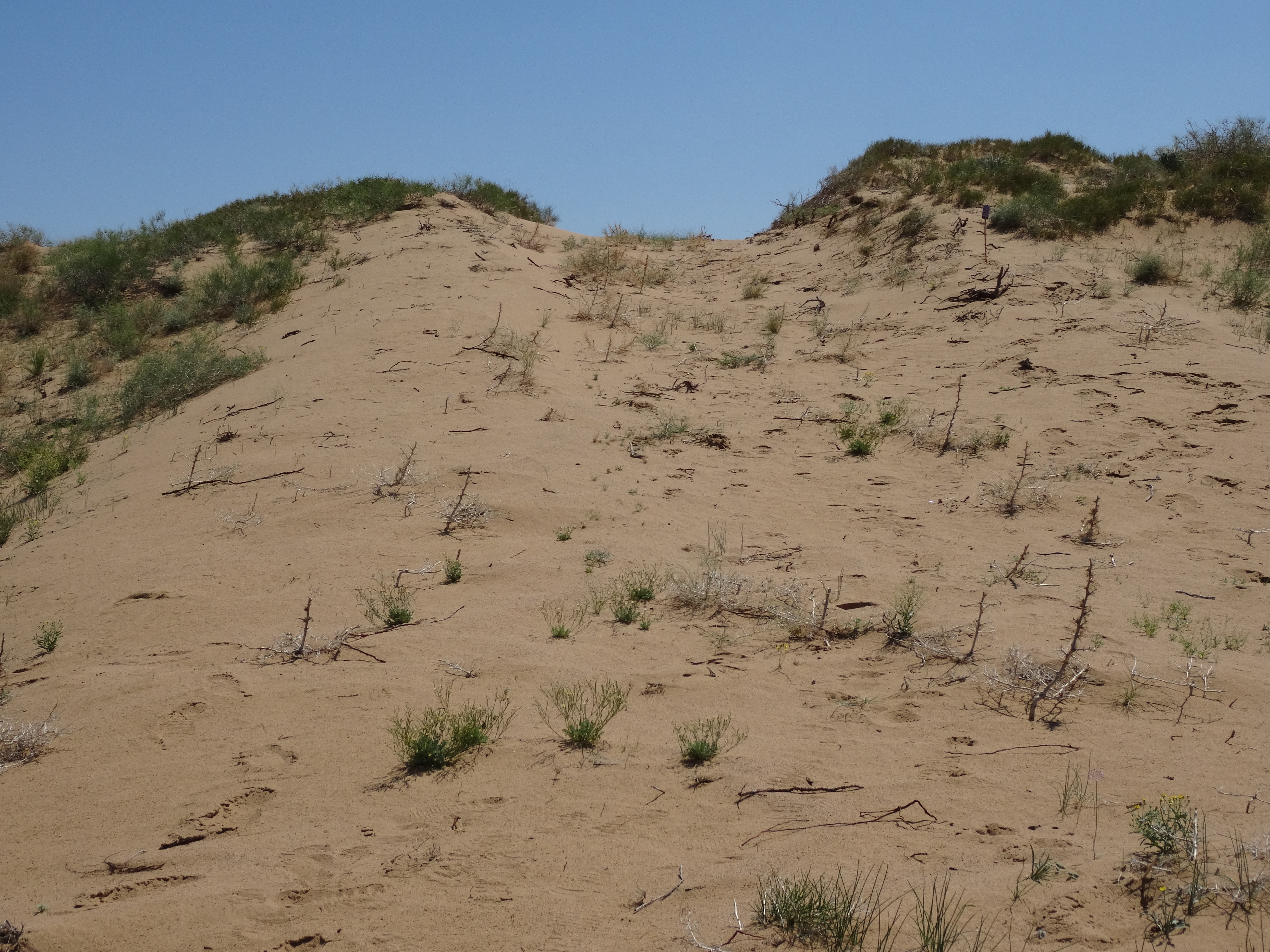

Supplement: Supplemental Information 1 — (A) Population. (B) Individual plant. (C) Flower characteristic. EP, external petal; LL, lateral lobe; S, stigma; ML, middle lobe. (D) Capsule with seeds showing longitudinal dehiscence. [file peerj-07-7066-s001.zip › Dataset of figure 1/figure 1-A.JPG]

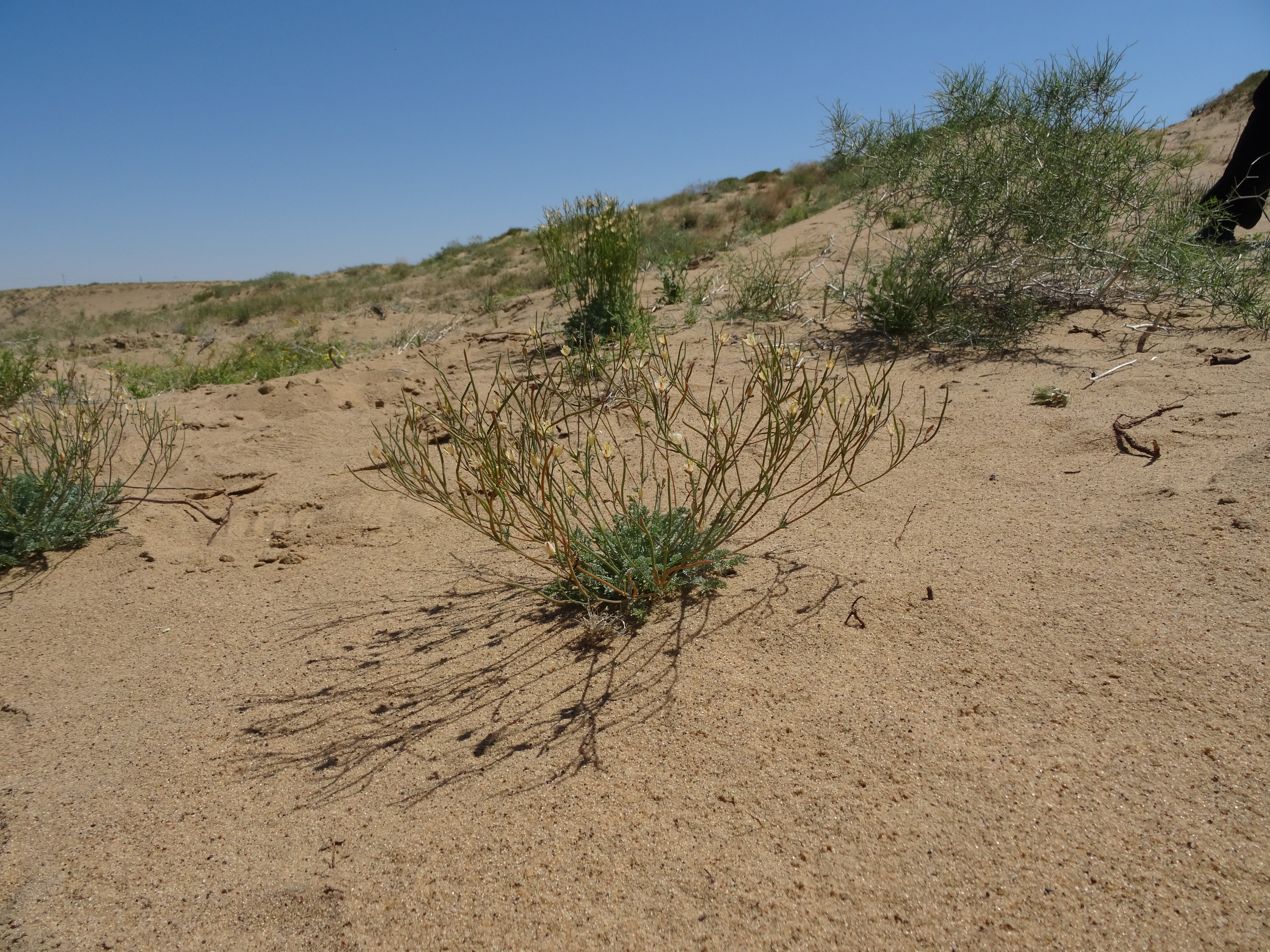

Supplement: Supplemental Information 1 — (A) Population. (B) Individual plant. (C) Flower characteristic. EP, external petal; LL, lateral lobe; S, stigma; ML, middle lobe. (D) Capsule with seeds showing longitudinal dehiscence. [file peerj-07-7066-s001.zip › Dataset of figure 1/figure 1-B.JPG]

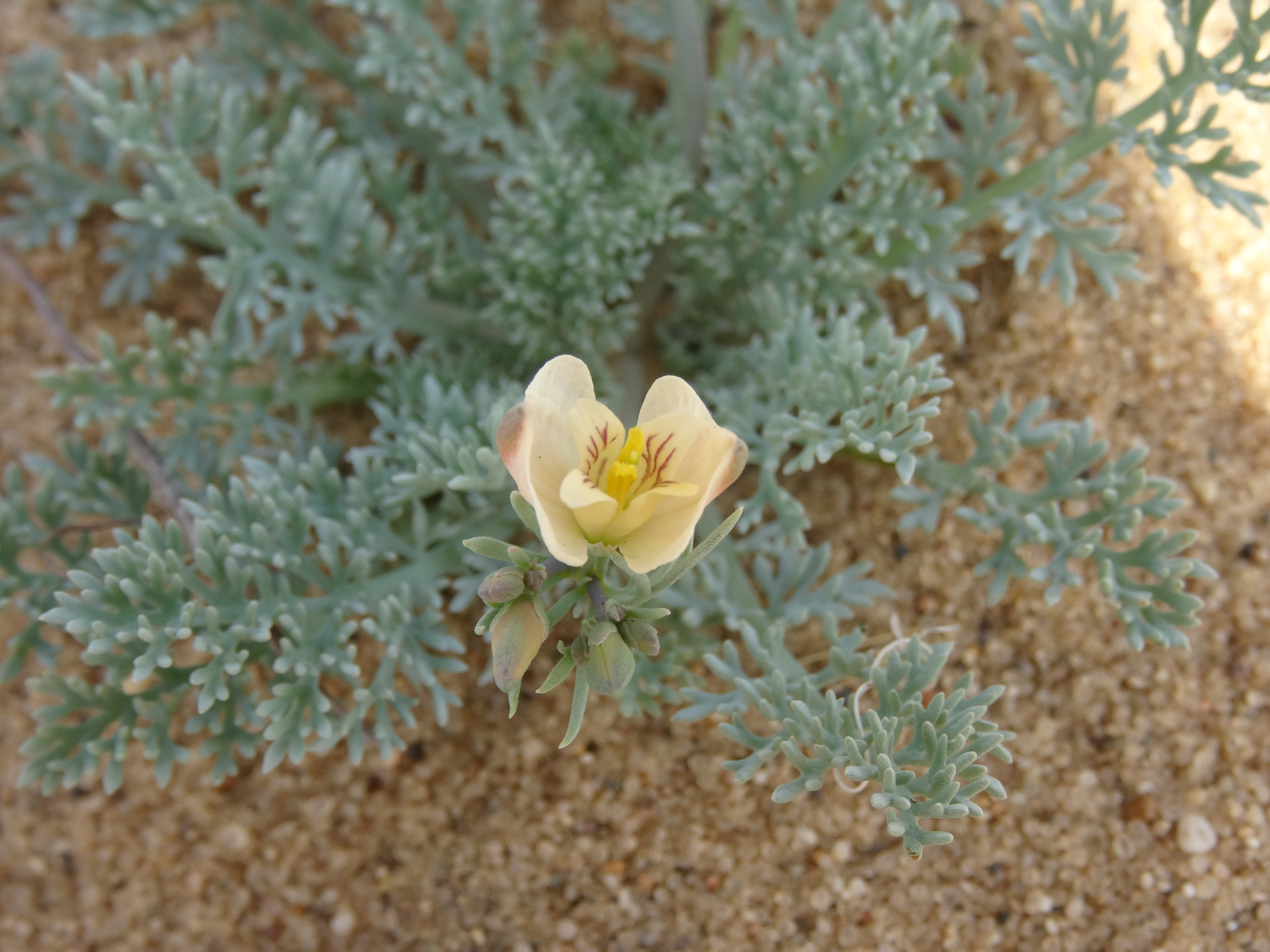

Supplement: Supplemental Information 1 — (A) Population. (B) Individual plant. (C) Flower characteristic. EP, external petal; LL, lateral lobe; S, stigma; ML, middle lobe. (D) Capsule with seeds showing longitudinal dehiscence. [file peerj-07-7066-s001.zip › Dataset of figure 1/figure 1-C.JPG]

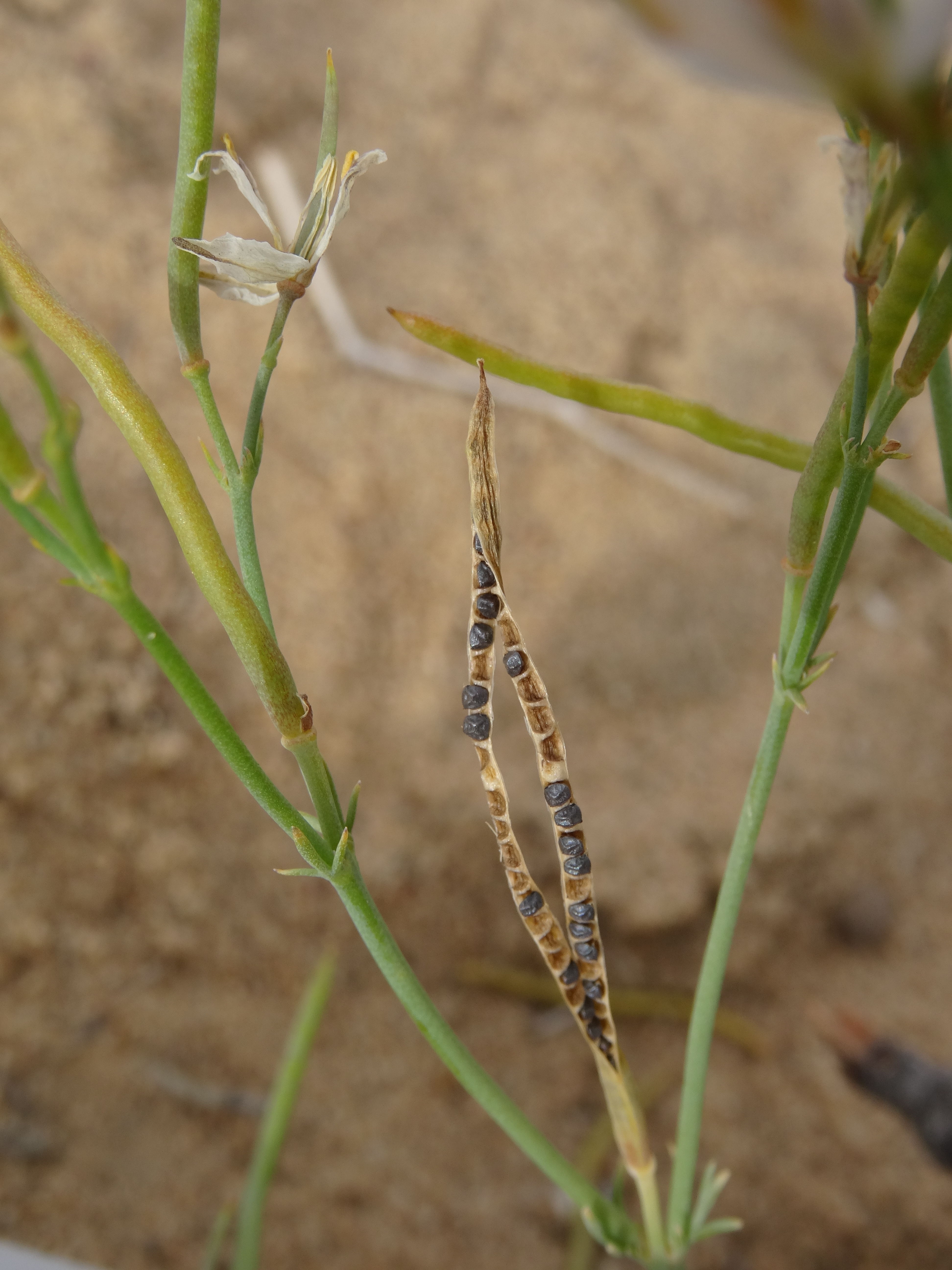

Supplement: Supplemental Information 1 — (A) Population. (B) Individual plant. (C) Flower characteristic. EP, external petal; LL, lateral lobe; S, stigma; ML, middle lobe. (D) Capsule with seeds showing longitudinal dehiscence. [file peerj-07-7066-s001.zip › Dataset of figure 1/figure 1-D.jpg]

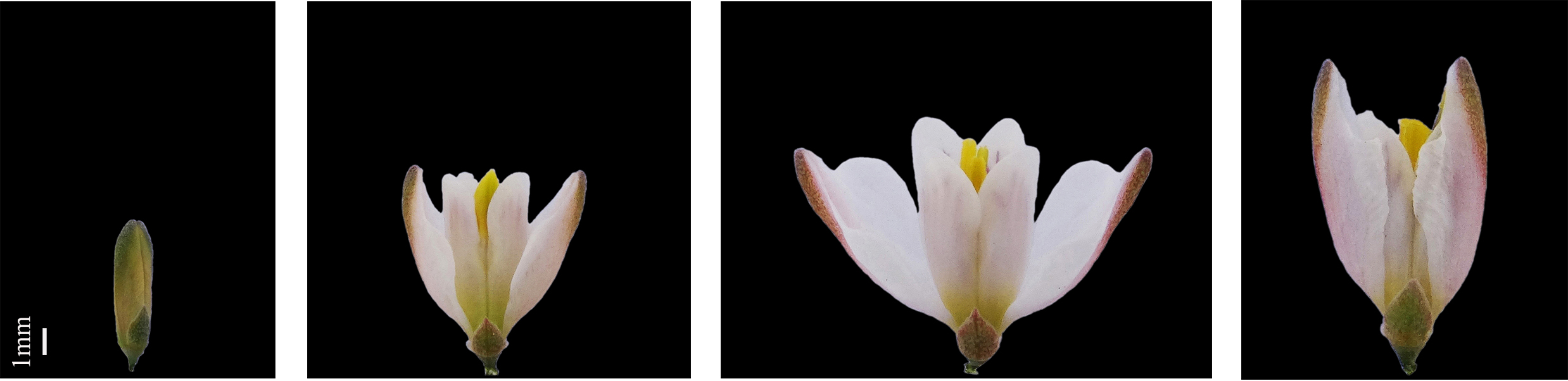

Supplement: Supplemental Information 2 — (Note: Different lowercase letters indicate significant differences among the same phase at p < 0.05 levels). [file peerj-07-7066-s002.zip › Dataset of figure 2/figure 2-a .jpg]

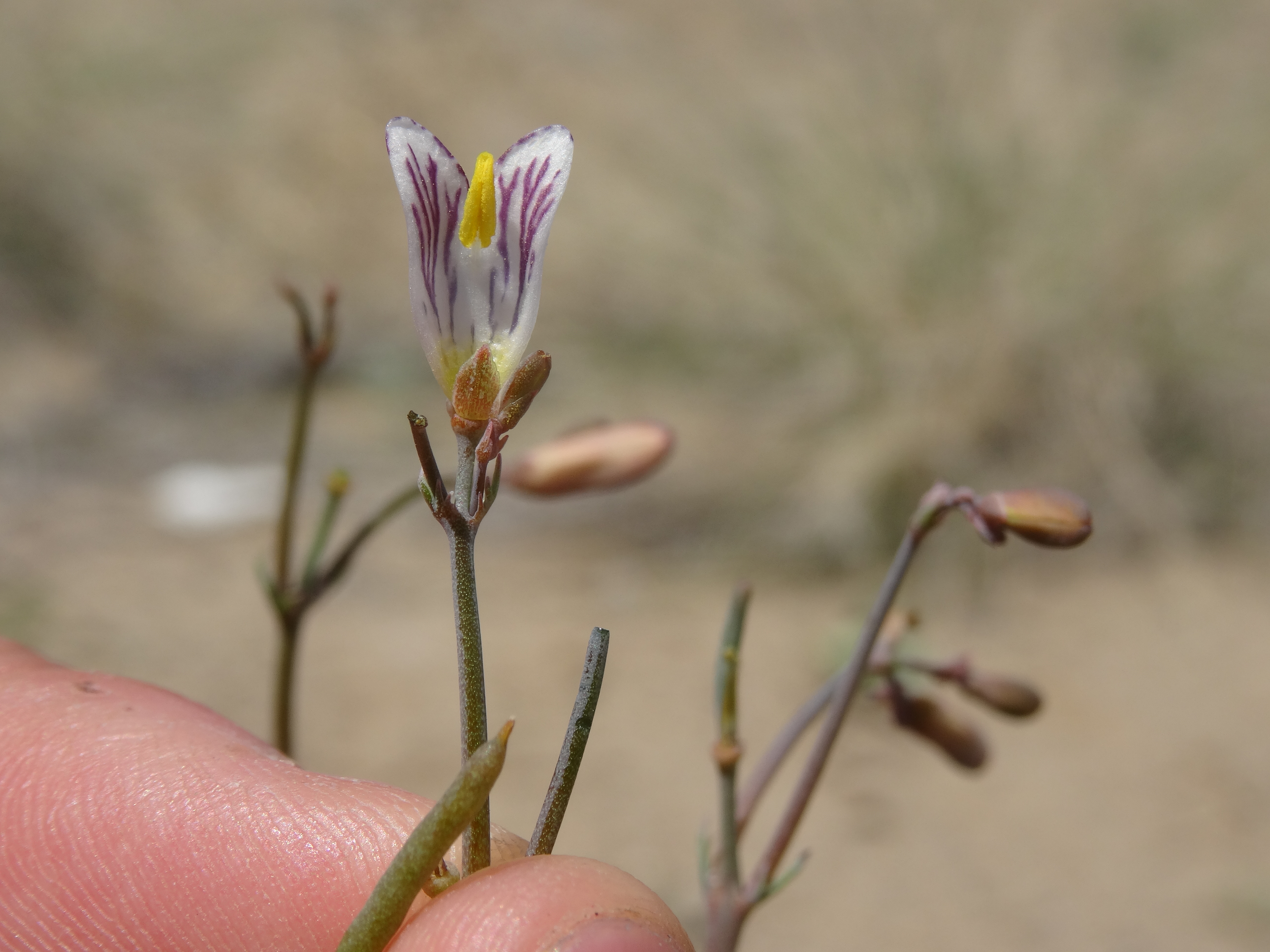

Supplement: Supplemental Information 3 — (A) After anthers dehiscence, most of the pollen grains were wrapped in the pouched middle lobe; (B) Three hours after middle lobes wrapped pollen, the middle lobe started to curl outward from the edge to form a gap and release a part of the pollen; (C) Eight hours after flowers start curling outward, the middle lobe is deflexed backward and releases all the pollen (the picture shows the back of the inner petal). [file peerj-07-7066-s003.zip › Dataset of figure 3/figure 3-B.JPG]

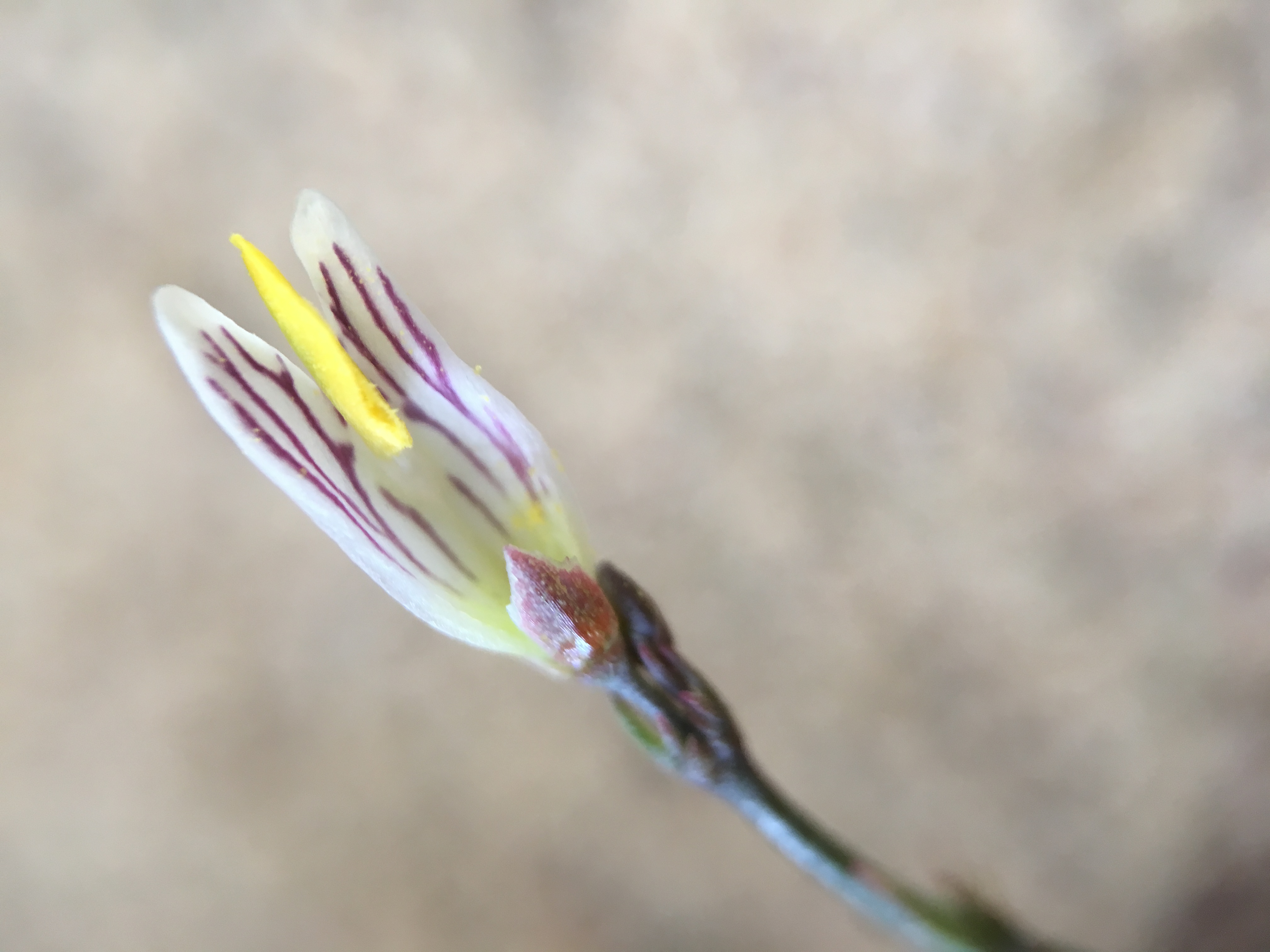

Supplement: Supplemental Information 3 — (A) After anthers dehiscence, most of the pollen grains were wrapped in the pouched middle lobe; (B) Three hours after middle lobes wrapped pollen, the middle lobe started to curl outward from the edge to form a gap and release a part of the pollen; (C) Eight hours after flowers start curling outward, the middle lobe is deflexed backward and releases all the pollen (the picture shows the back of the inner petal). [file peerj-07-7066-s003.zip › Dataset of figure 3/figure 3-A.JPG]

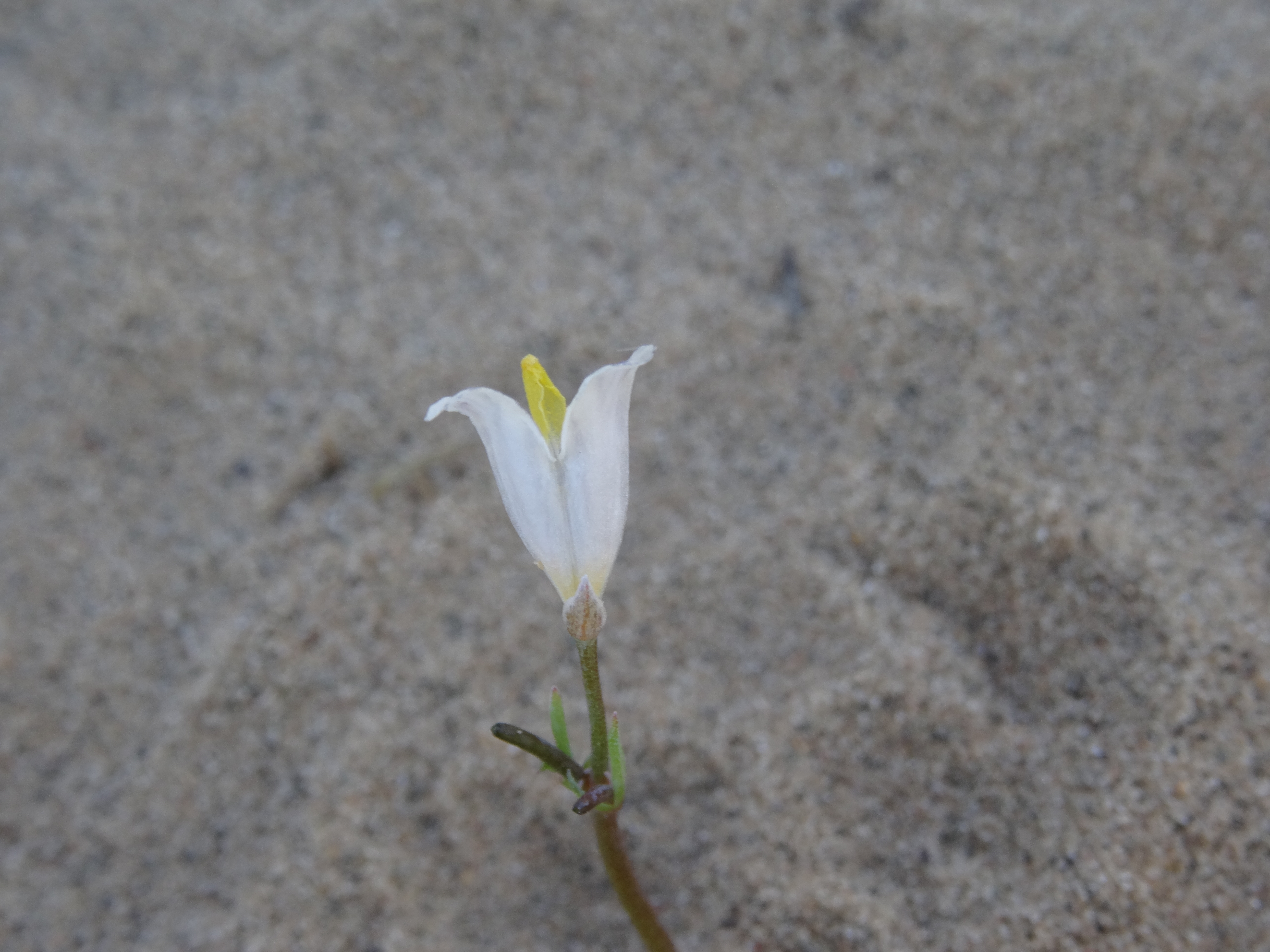

Supplement: Supplemental Information 3 — (A) After anthers dehiscence, most of the pollen grains were wrapped in the pouched middle lobe; (B) Three hours after middle lobes wrapped pollen, the middle lobe started to curl outward from the edge to form a gap and release a part of the pollen; (C) Eight hours after flowers start curling outward, the middle lobe is deflexed backward and releases all the pollen (the picture shows the back of the inner petal). [file peerj-07-7066-s003.zip › Dataset of figure 3/figure 3- C.JPG]

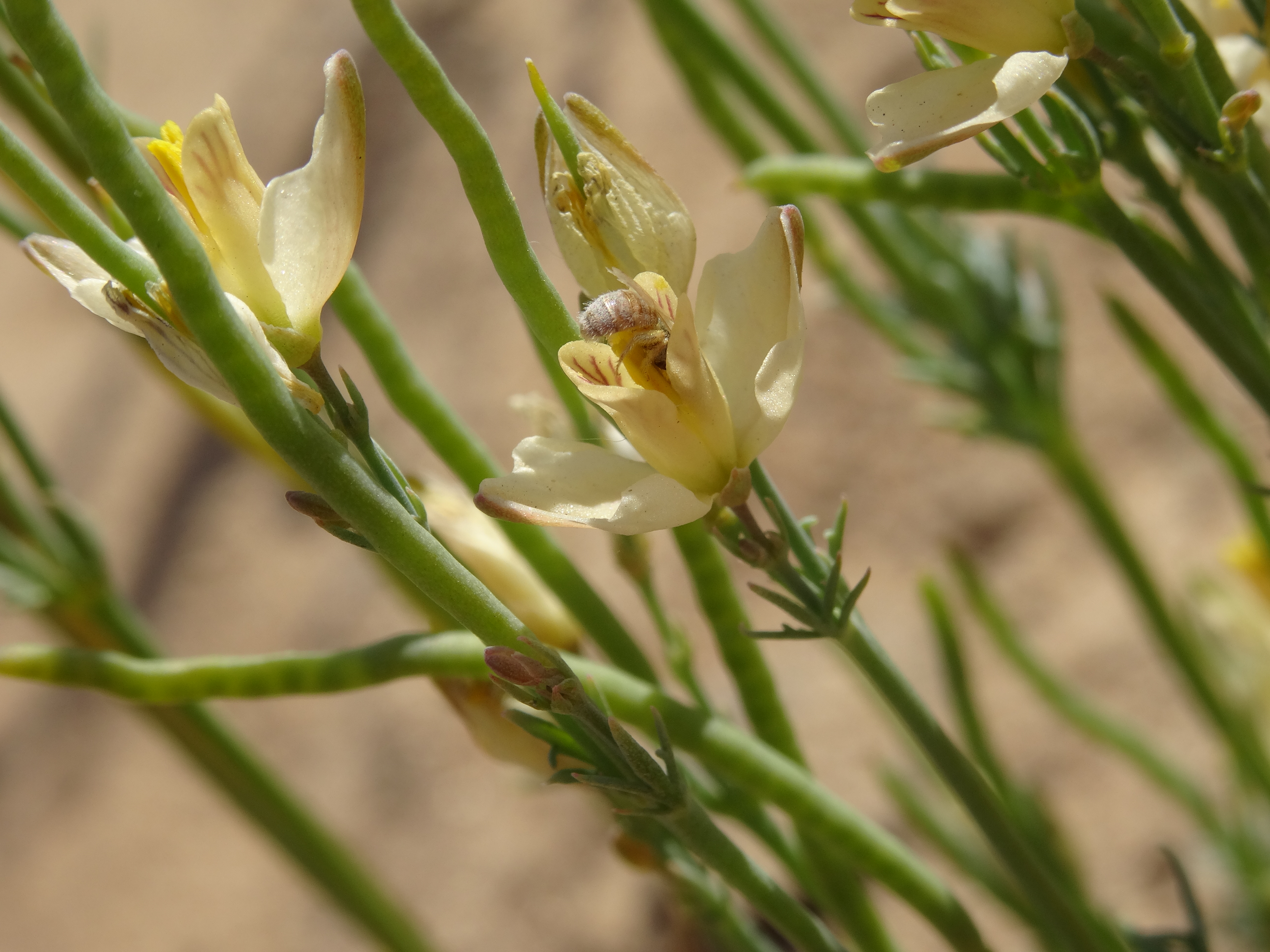

Supplement: Supplemental Information 5 — (A) Foraging behavior I: pollinator collects the pollen from the closed middle lobes; (B) Foraging behavior II: pollinator sticking its body into the corolla to suck nectar when middle lobe deflexed. [file peerj-07-7066-s005.zip › Dataset of figure 5/figure 5-B.JPG]

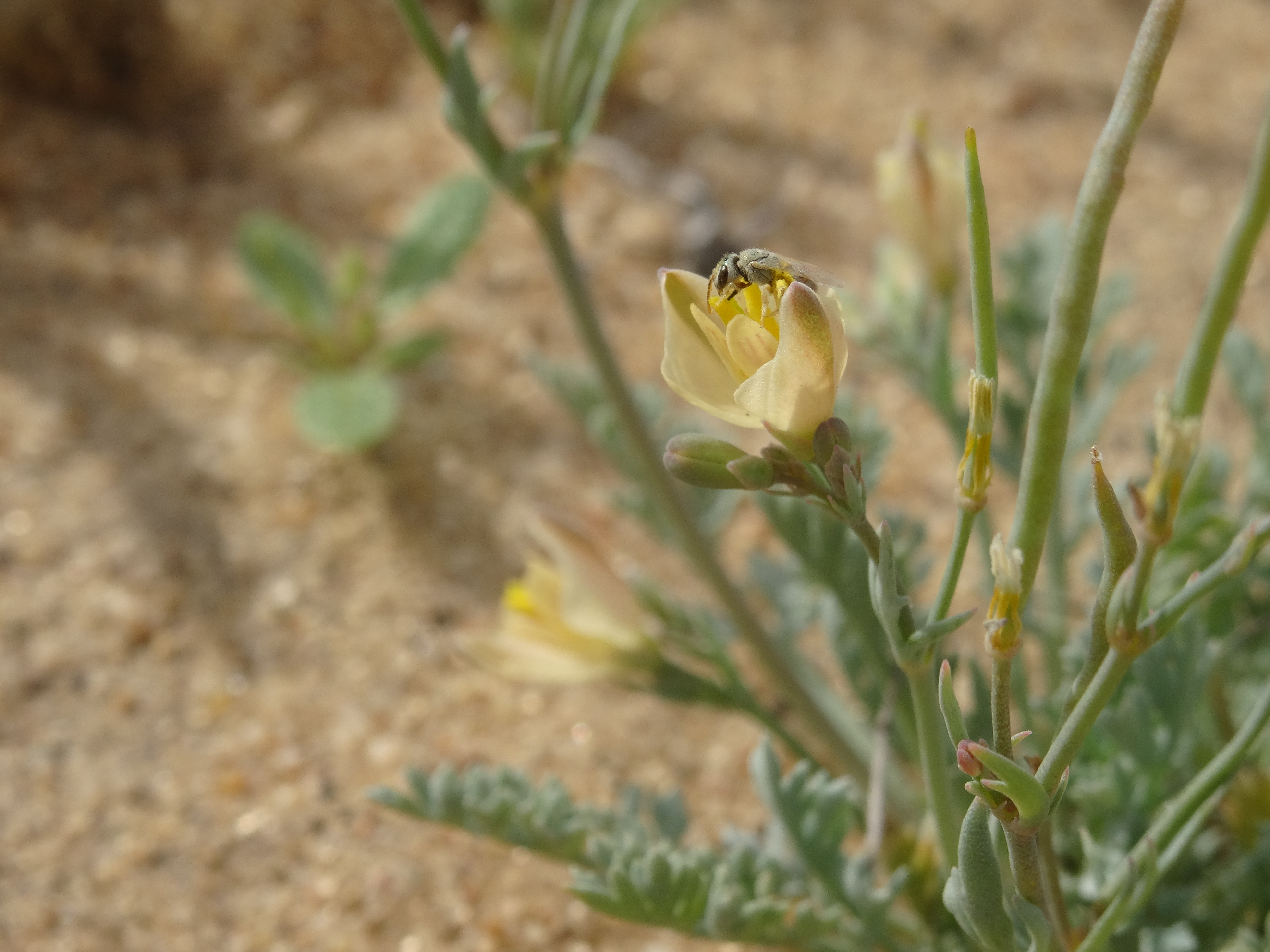

Supplement: Supplemental Information 5 — (A) Foraging behavior I: pollinator collects the pollen from the closed middle lobes; (B) Foraging behavior II: pollinator sticking its body into the corolla to suck nectar when middle lobe deflexed. [file peerj-07-7066-s005.zip › Dataset of figure 5/figure 5-A.JPG]
